# Significant Multi Tesla Fields Within a Solenoid Encircled by Nanostructure Windings

**DOI:** 10.1038/s41598-018-38306-8

**Published:** 2019-02-28

**Authors:** C. R. (Bob) James, John E. Long, Dwight E. Manning

**Affiliations:** 1 Professor Emeritus University of Alberta and President, 1994680 Alberta Limited, Calgary, Canada; 2 Chairman of the Board of 1994680 Alberta Limited, Calgary, Canada; 3 Secretary of 1994680 Alberta Limited, Calgary, Canada

## Abstract

This theoretical investigation develops analytical concepts on how to realize magnetic fields for the Multi to Mega Tesla realm within the range from milli to nano volumes and periods. The fields are anticipated to be realized using solenoids with multi-walled carbon (MWCNT) nanostructure composite metal windings. The study presented here opens up the issue of the miniaturization of large magnetic field systems. Concern has been raised that for such large magnetic fields and associated energies that the solenoidal structure may be highly reconfigured. Consequently, an investigation is undertaken of the tensile strength resulting from the azimuthal current. Also, the azimuthal power per turn will be addressed along with its limitations in generating the magnetic field. Further, the study finds the allowable eigenvalue frequencies of the electric field. Tables are provided for given values of *B*_*z*0_(0) from 2*T* to 2(10)^6^*T* for a number of important parameters for consideration in designing solenoidal systems.

## Introduction

This theoretical investigation develops analytical concepts on how to realize magnetic fields for the Multi to Mega Tesla realm within the range from milli to nano volumes and periods. The fields are anticipated to be realized using solenoids with multi-walled carbon composite (MWCNT) metal nanostructure windings, Subramanian, *et al*.^1^. This publication on multi-walled carbon nanotube (MWCNT) – copper composite material provides experimental evidence on the properties of the material including the current carrying capacity capability which was measured at 6(10)^12^*A*/*m*^2^. Also support of increased current carrying capacity has been measured by^2,3^. The presentation herein has recognized these experimental results in MWCNT – Copper composite material and has adopted the results of Subramanian, *et al*.^1^ in the examples presented in Charts 1 and 2.

A major examination of the electrical properties of carbon nanotubes and their future use in electrical systems is given by Agnieszka Lekawa-Raus, *et al*.^4^. In^5^ a study of a strategy on improving the interface between copper and carbon nanotubes (CNT) gives a calculation of a current density of 10^15^*A*/*m*^2^. Also, studies are given on electrical properties of CNT’s^6,7^. In the paper^8^, it is noted that CNT’s have extremely high current carrying capacity of one thousand times higher than that of copper^9^. In^8^, examination is undertaken to represent the fabrication of copper/single-walled carbon nanotubes (SWCNT) composite film with homogeneous dispersed nanotubes by electroless disposition. As stated in that paper the properties such as the electrical and thermal conductivity, ampacity, and SWCNT content will be evaluated in future work. The study presented here opens up the issue of the miniaturization of large magnetic field systems. This in turn might potentially lead to applications on medical, solenoidal, transformer and motor technology. The results presented may assist in the understanding of the role of the magnetic field strength required in magnetocompression aiding a laser ignition fusion machine. Prior to the laser ionization of the target material, one role is having the potential of acting as a precursor to ignition of the target consisting of deuterium/tritium atoms by precompressing the target and increasing its binding energy. Concern has been raised that for such large magnetic fields and associated energies that the solenoidal structure may be highly reconfigured. Some understanding of the issue might be gained by noting from V. Canuto and D.C. Kelly^10^ where they show that for hydrogen, fields in excess of 4.7(10)^5^*T* modifications of characteristic atomic scales of length and binding energy becomes noteworthy. When increasing such a field, quantum mechanics shows that the orbit of a hydrogen atoms electron has some elongation in the direction of a strong magnetic field as shown by Aringazin^11^. A significant stress is experience by the windings of the solenoidal structure from the tensile strength resulting from the azimuthal current flowing in the coils generating the axial magnetic field. This tensile strength will be investigated in the following text. As well, the azimuthal power per turn will be addressed along with its limitations in generating the magnetic field. Further, the study will find in the analysis the resulting eigenvalue frequencies. Their limitations are considered.

In the examples considered, a constant magnetic field is assumed inside the solenoidal winding. By using the angular momentum expression given in the amended Bohr model, the orbital period, *τ*, of the ground state electron for hydrogen and its isotopes is

$$\tau \,=\,\frac{2\pi {r}_{eo}}{{v}_{eo}}\,=\,\frac{2\pi {m}^{\ast }}{\frac{h}{2\pi {r}_{eo}^{2}}\,+\,\frac{1}{2}eB}$$

where *v*_*eo*_ = electron velocity, *r*_*eo*_ = electron orbital radius and m^*^ = mass of the electron. For *B* = *O*

$$\tau \,=\,1.52\,{(10)}^{-16}\,s$$

and for the largest field considered in this text of 2(10)^6^*T*

$$\tau \,=\,1.54\,{(10)}^{-17}\,s$$

Consequently, for any signal with a period much greater than (10)^−16^ *s*, the magnetic field seen by the electron approximates a constant. In the examples considered in this text the smallest signal period is of the order (10)^−11^ *s*.

This information provides some insight on how molecular structure behaves when immersed in large magnetic fields. For material such as found in an on axis fusion target when exposed to the magnetic field, the electric field is not of consequence as will be shown because the axial electric field is zero.

The current world record for the highest generated magnetic field claimed by the National High Magnetic Field Laboratory is 100.75 T^12^. The material herein offers results to assist in luminating a number of the ongoing magnetic field issues to be examined. Tables are provided for given values of B_***z***0_(0) from 2T to 2(10)^6^ T for the cases of first order and fourth order eigenvalues of the electric field. In each case the values of the current density in the coil, the loop current, the voltage drop in one turn of the coil, the average power delivered per turn, the tensile strength on the outer surface per turn, and the solenoidal energy density stored within the solenoid per unit length are presented. Specifically, when considering prototypes, the potential parameters for assessing the theory are presented for large magnetic field solenoidal systems with multi-walled carbon nanotube (MWCNT) – copper composite windings where *B*_*zO*_(0) = *2; 20; 200; 2000; 20,000; 200,000; and 2,000,000* *T*. Magnetic fields in the range of 2000 *T* or less are shown to produce tensile strengths within the current range of such strengths for carbon-nano-tubes. However, for extremely short periods like some analyzed, the effective tensile strength may be increased due the inability of material to adjust.

The presentation provides theoretical values for magnetic field densities that may be speculated for consideration for solenoidal systems.

## A Solenoidal B Field Model – A Cylindrical System

The vectors used in the analysis are defined as:

*B* = *the magnetic field density vector*, *H* = *the magnetic field intensity vector*,

*E* = *the electric field intensity vector*, *D* = *the electric field intensity vector*

*j* = *the electric current surface density*, *ρ* = *volume electric charge density*,

where *B* = *μ H and D* = *ε E and* the physical constants being

*μ* = *the magnetic permeability*,

*ε* = *the electric permittivity*

All the quantities used in the analysis will be in Mks units.

Assumptions are made that *B*_*r*_ = *B*_*θ*_ = *E*_*z*_ = *E*_*r*_ = *j*_*r*_ = *j*_*z*_ = *ρ* = 0

and only *r* and *t* dependence. The current *j*_*θ*_ flows around the solenoid with a diameter *d* = 2*r*

where *r* is the radius.

**Figure 1:**
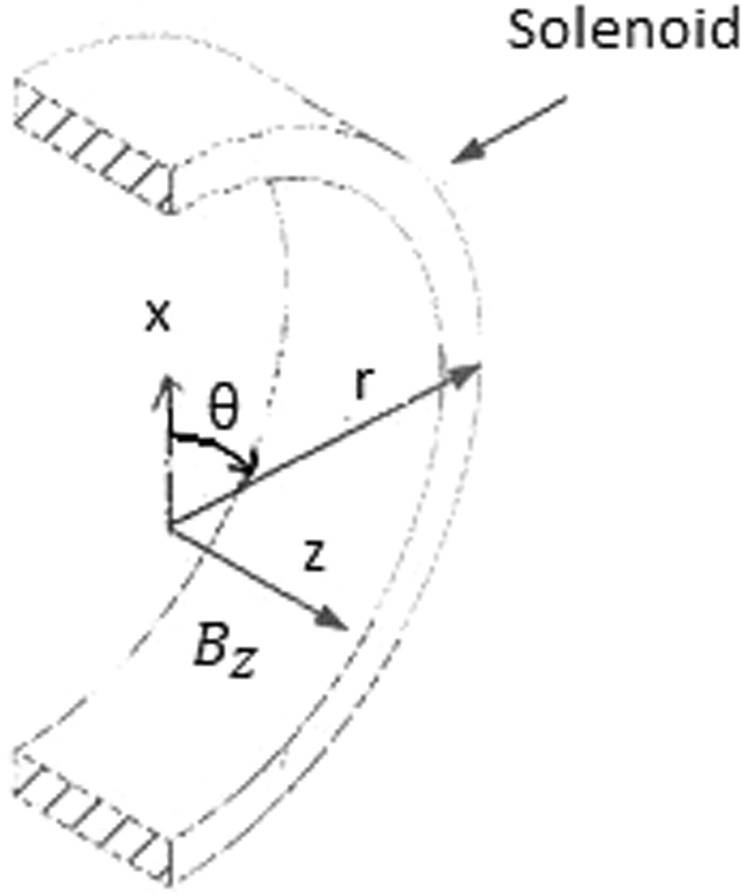
The coordinates r-θ-z and the magnetic field vector *B*_*Z*_ are illustrated.

From Maxwell’s equations in cylindrical coordinates (as shown in Fig. 1)

1 $$\frac{1}{r}\frac{\partial }{\partial r}(r{E}_{\theta })\,=\,-\,\,\frac{\partial {B}_{z}}{\partial t}$$

2 $$-\frac{\partial {H}_{z}}{\partial r}\,=\,{j}_{\theta }\,+\,\,\frac{\partial {D}_{\theta }}{\partial t}$$

For 0 ≤ *r* < $\frac{d}{2}$ with *j*_*θ*_ = 0 equation (2) becomes

$$-\frac{\partial {H}_{z}}{\partial r}\,=\,\,\frac{\partial {D}_{\theta }}{\partial t}$$

and from (1)

3 $$\frac{\partial }{\partial r}\,[\,\frac{1}{r}\,\frac{\partial }{\partial r}\,(r{E}_{\theta })]\,=\,\mu \varepsilon \,\frac{{\partial }^{2}{E}_{\theta }}{\partial {t}^{2}}$$

By assuming the time dependence *e*^−*iωt*^ and *μ* = *μ*_0_ = 1.257(10)^−6^*henry*/*m*, (see^13^ Kaye and Laby) then from (3)

$$\frac{\partial }{\partial r}\,[\frac{1}{r}\,\frac{\partial }{\partial r}\,(r{E}_{\theta })]\,=\,-\,{\omega }^{2}\,{\mu }_{0}\varepsilon \,{E}_{\theta }$$

and with *E*_*θ*_ = *A*_*θ*_*e*^−*iωt*^, which satisfies the Bessel differential equation, the solution for *A*_*θ*_ is

$${A}_{\theta }\,=\,{C}_{1}\,{J}_{1}\,(\omega \sqrt{{\mu }_{0}\varepsilon }r)\,+\,{C}_{2}\,{N}_{1}(\omega \sqrt{{\mu }_{0}\varepsilon }r)$$

where ${J}_{1}\,(\omega \sqrt{{\mu }_{0}\varepsilon }r)$ is the Bessel Function of the First Kind and ${N}_{1}\,(\omega \,\sqrt{{\mu }_{0}\varepsilon }r)$ is the Bessel Function of the Second Kind

Since *E*_*θ*_ must be finite for *r* = 0 and *N*_1_(0) → ∞, then *C*_2_ = 0 Hence,

4 $${A}_{\theta }\,=\,{C}_{1}\,{J}_{1}\,(\omega \,\sqrt{{\mu }_{0}\varepsilon }r)$$

At *r* = $\frac{d}{2}$ by assuming a perfect conducting boundary,

5 $${A}_{\theta }(\frac{d}{2})\,=\,{C}_{1}\,{J}_{1}\,(\frac{\omega \sqrt{{\mu }_{0}\varepsilon }d}{2})\,=\,0$$

Therefore,

6 $${\omega }_{l}\,\sqrt{{\mu }_{0}\varepsilon }\,d\,=\,2{\delta }_{l}$$

where *δ*_*l*_ is the *l*^th^ root of

$${J}_{1}(\frac{\omega \sqrt{{\mu }_{0}\varepsilon }d}{2})\,=\,0$$

and $${\delta }_{l}\,=\,3.832,\,7.016,\,10.173,\,13.3\,\ldots $$

From (1)

$${B}_{z}\,=\,(\frac{1}{i\omega r})[r\,\frac{\partial {E}_{\theta }}{\partial r}\,+\,{E}_{\theta }]$$

and since

$${E}_{\theta }\,=\,{C}_{1}\,{J}_{1}\,(\omega \sqrt{{\mu }_{0}\varepsilon }r)\,{e}^{-i\omega t}$$

Then

7 $${B}_{z}\,=\,\frac{{{\rm{C}}}_{1}}{i\omega }[\,\frac{d{J}_{1}}{dr}\,+\,\frac{1}{r}\,{J}_{1}]\,{e}^{-i\omega t}$$

from the derivative expression for Bessel Functions

$${B}_{z}\,=\,\sqrt{{\mu }_{0}\varepsilon }\,\frac{{C}_{1}}{i}[{J}_{0}(\omega \sqrt{{\mu }_{0}\varepsilon }r)]\,{e}^{-i\omega t}$$

or

8 $${B}_{z}\,=\,\sqrt{{\mu }_{0}\varepsilon }\,\frac{{C}_{1}}{i}\,{J}_{0}(\omega \sqrt{{\mu }_{0}\varepsilon }r)\,{e}^{-i\omega t}$$

From (4 and 8) with evaluating *B*_*Z*_ at *r* = 0 and *t* = 0 which is a constant magnetic field defined as *B*_*z*0_. Hence, from (8).

$$\frac{{C}_{1}}{i}\,=\,\,\frac{{B}_{z0}(0)}{\sqrt{{\mu }_{0}\varepsilon }}$$

and,

9 $${B}_{z}(r,t)\,=\,{B}_{z0}(0){J}_{0}(\omega \sqrt{{\mu }_{0}\varepsilon }r){e}^{-i\omega t}$$

10 $${E}_{\theta }(r,t)\,=\,\,\frac{i{B}_{z0}(0)}{\sqrt{{\mu }_{0}\varepsilon }}\,{J}_{1}\,(\omega \sqrt{{\mu }_{0}\varepsilon }r){e}^{-i\omega t}$$

The perfect conductor assumption approximation is based upon first noting the values measured by Subramanian, *et al*.^1^ for their carbon-copper nanotubes are *j*_*θ*_ = 6(10)^12^*A*/m^2^ and the conductivity *σ* = 4.7(10)^7^ s/m. By using these values, from Ohm’s Law, the electric field in the windings of the coil enclosing the solenoid is 1.3 (10)^5^ V/m. Secondly, in (10) the least favorable case is for *B*_*z*0_(0) = 2 T. Also, in (10)

(i) the first order field eigenvalue mode equals 3.832^14,15^ and the maximum of $\,{J}_{1}(\omega \sqrt{{\mu }_{0}\varepsilon }r)$ occurs at 0.58. The corresponding |*E*_*θ*_| = 3.5(10)^8^ V/m which is more than a thousand times greater than the coil electric field. Further, for $\omega (\sqrt{{\mu }_{0}\varepsilon }r)\,=\,3.8$, $\,{J}_{1}(\omega \sqrt{{\mu }_{0}\varepsilon }r)\,=\,0.0128$ and so |*E*_*θ*_| = 0.45(10)^7^ V/m which is 35 times greater than the coil electric field. As well, this evaluation gives a radial distance that is 99% of the eigenvalue distance at the coil. The transition from the perfect conductor role to the solenoid electric field is extremely short, a step.
(ii) the fourth order field eigenvalue equals 13.324 and the maximum of ${J}_{1}(\omega \sqrt{{m}_{0}\varepsilon }r)$ occurs at 0.23. The corresponding |*E*_*θ*_| = 0.8(10)^8^ V/m which is over 600 times greater than the coil electric field. Furthermore, for $\omega (\sqrt{{\mu }_{0}\varepsilon }r)=13.2$, ${J}_{1}\,(\omega \sqrt{{m}_{0}\varepsilon }r)\,=\,0.0271$ and so |*E*_*θ*_| = 2.168(10)^6^ V/m which is 17 times greater than the coil electric field. As well, the above evaluation gives a radial distance that is 99% of the eigenvalue distance at the coil.

From this information the perfect conductor assumption approximation appears appropriate for the analysis given since over 99% of the radial distance the effect of the electric field in the windings of the coil is negligible for the study.

What is envisioned is that |*E*_*θ*_| is approximated by the perfect conductor solution inside the solenoid cavity. At the radius of the solenoid determined by the field eigenvalue the electric field in the windings of the coil will be represented by the step function times the electric field determined by Ohm’s Law.

## The Magnetic Field Boundary Condition

In the conducting material from $r\,=\,\frac{d}{2}\,{\rm{to}}\,\frac{d}{2}\,+\,\lambda$ the displacement current from (2) can be neglected since from Ohm’s Law and using *σ* = 4.7(10)^7^ *s*/m found by Subramanian, *et al*.^1^ the current density contribution by the displacement current is

$$\frac{2\pi \,f\,\varepsilon }{\sigma }$$

and when *ε* = *ε*_0_ the aforementioned term is 1.2*f*(10)^−18^ which can be neglected for

*f* < 10^17^ *Hz*. Hence, by setting

$$\frac{\partial {D}_{\theta }}{\partial t}\,\approx \,0$$

and integrating (2) gives

11 $$-\,{\int }_{\frac{d}{2}}^{\frac{d}{2}+\lambda \,}\frac{\partial {B}_{z}}{\partial r}\,dr\,=\,{\mu }_{0}{\int }_{\frac{d}{2}}^{\frac{d}{2}+\lambda }{j}_{\theta }\,dr$$

where *μ* is set equal to *μ*_0_ since carbon and copper have such a value to five orders of magnitude^13^.

Here, for radial simplicity, *λ* is set equal to the radial thickness of the conducting material.

At the outer surface of the conducting material where, $r\,=\,\,\frac{d}{2}\,+\,\lambda$, the magnetic field is taken as zero.

It should be noted that a corresponding analysis should be followed in multi-walled nanotube windings between adjacent walls.

Hence, from (11)

12 $$\begin{array}{ccc}{B}_{z}(\frac{d}{2}) & = & {\mu }_{0}\,{\int }_{\frac{d}{2}}^{\frac{d}{2}+\lambda }{j}_{\theta }dr\\ {B}_{z}(\frac{d}{2}) & = & {B}_{z0}(\frac{d}{2})\,{e}^{-i\omega t}\end{array}$$

Next, by setting *j*_*θ*_ = *j*_*θ*0_*e*^−*iωt*^ where *j*_*θ*0_ = constant from (12)

13 $${B}_{z0}(\frac{d}{2})\,=\,{\mu }_{0}{j}_{\theta 0}\lambda $$

After returning to (8 and 13) at $\,\frac{d}{2}$,

$$\sqrt{{\mu }_{0}\varepsilon }\,{J}_{0}\,(\omega \sqrt{{\mu }_{0}\varepsilon }\frac{d}{2}\,)\frac{{C}_{1}}{i}\,=\,{\mu }_{0}{j}_{\theta 0}\lambda $$

Hence,

$$\frac{{C}_{1}}{i}\,=\,\,\frac{{\mu }_{0}\,\lambda \,{j}_{\theta 0}}{\sqrt{{\mu }_{0}\varepsilon }\,{J}_{0}(\omega \,\sqrt{{\mu }_{0}\varepsilon }\frac{d}{2})}$$

and from (8)

14 $${B}_{z}\,=\,{\mu }_{0}\,{j}_{\theta 0}\,\lambda \,\frac{\,{J}_{0}(\omega \,\sqrt{{\mu }_{0}\varepsilon }\,r)}{\,{J}_{0}(\omega \,\sqrt{{\mu }_{0}\varepsilon }\frac{d}{2})}\,{e}^{-i\omega t}.$$

For *r* = 0 and by using (13 and 14), and *B*_*z*_ = *B*_*z*0_(0)*e*^−*iωt*^

$${B}_{z0}(0)\,=\,\frac{{\mu }_{0}\,\lambda \,{j}_{\theta 0}}{\,{J}_{0}(\omega \,\sqrt{{\mu }_{0}\varepsilon }\frac{d}{2})}$$

with from (13)

$${B}_{z0}(\frac{d}{2})\,=\,{J}_{0}(\omega \sqrt{{\mu }_{0}\varepsilon }\frac{d}{2})\,{B}_{z0}\,(0).$$

Hence,

15 $${j}_{\theta 0}\,=\,\,\frac{{B}_{z0}\,(0){J}_{0}(\omega \,\sqrt{{\mu }_{0}\varepsilon }\frac{d}{2})}{{\mu }_{0}\lambda }.$$

On returning to (12) and noting that the differential current is

$$d{I}_{\theta }{e}^{-i\omega t}={j}_{\theta }dzdr,\,{\rm{o}}{\rm{r}}\,d{I}_{\theta }={j}_{\theta 0}dz\,dr$$

then for *B*_*z*_$(\frac{d}{2})$ = *B*_*z*0_$(\frac{d}{2})$ *e*^−*iωt*^ from (12)

it is found that

$${B}_{z0}\,(\frac{d}{2})\,=\,{\mu }_{0}\,{\int }_{\frac{d}{2}}^{\frac{d}{2}+\lambda }{j}_{\theta 0}dr$$

$${I}_{\theta }\,=\,{\int }_{0}^{L}dz\,{\int }_{\frac{d}{2}}^{\frac{d}{2}+\lambda }{j}_{\theta 0}\,dr\,=\,L\,{\int }_{\frac{d}{2}}^{\frac{d}{2}+\lambda }{j}_{\theta 0}dr$$

where L = length of the solenoid. Hence,

16 $$\begin{array}{ccl}{B}_{z0}(\frac{d}{2}) & = & {\mu }_{0}\,\,\frac{{I}_{\theta }}{L}\\  & = & {J}_{0}\,(\omega \sqrt{{\mu }_{0}\varepsilon }\frac{d}{2})\,{B}_{z0}\,(0)\end{array}$$

and for *N* current loops existing in length *L* with each loop current represented by *I*_*θN*_, then

$${I}_{\theta }=N{I}_{\theta N}$$

Consequently,

$${B}_{z0}\,(\frac{d}{2})\,=\,{\mu }_{0}\,\frac{N}{L}\,{I}_{\theta N}$$

By defining for example, the axial thickness of the conducting material as

$${\rm{\Delta }}\,=\,\frac{L}{N},$$

$${B}_{z0}\,(\frac{d}{2})\,=\,\frac{{\mu }_{0}}{{\rm{\Delta }}}\,{I}_{\theta N}$$

or

$${J}_{0}\,(\omega \,\sqrt{{\mu }_{0}\varepsilon }\frac{d}{2}){B}_{z0}(0)\,=\,\frac{{\mu }_{0}}{{\rm{\Delta }}}\,{I}_{\theta N}$$

Hence,

17 $${I}_{\theta N}\,=\,{\rm{\Delta }}\,\frac{{J}_{0}\,(\omega \,\sqrt{{\mu }_{0}\varepsilon }\,\frac{d}{2})\,{B}_{z0}\,(0)}{{\mu }_{0}}$$

Also,

18 $${I}_{\theta N}\,=\,\,\frac{1}{N}\,{I}_{\theta }\,=\,\,\frac{L}{N}{\int }_{\frac{d}{2}}^{\frac{d}{2}+\lambda }{j}_{\theta 0}dr\,=\,{\rm{\Delta }}\,\,{j}_{\theta 0}\,\lambda $$

## Voltage ***V***_***T***_ and Average Power P

The derivation of an expression for the voltage, *V*_*T*_, can begin with Ohm’s Law which gives an average electric field in the composite wire carrying the current flowing around the solenoid. By using Ohm’s Law, an approximation of the average electric field can be set as *j*_*θ*0_/*σ* where *σ* is the conductivity of the wire. Consequently, the average voltage drop, *V*_*T*_, in one turn of the composite wire is found by multiplying the average electric field by *πd*.

Hence,

19 $${V}_{T}\,=\,\frac{\,{j}_{\theta 0}\,\pi d}{\sigma \,}$$

If the one turn is made from M equal segments, each segment has a voltage of *V*_*T*_/*M*. The approximate average power, delivered per turn by using the substitution of (15 and 17) is

20 $$P\,=\,{I}_{\theta N}\,{V}_{T}\,=\,\frac{{\rm{\Delta }}\pi d}{\lambda \sigma }{[\frac{{J}_{0}(\omega \sqrt{{\mu }_{0}\varepsilon }\frac{d}{2}){B}_{z0}(0)}{{\mu }_{0}}]}^{2}$$

## Tensile Strength

The radial tensile strength of one revolution of the solenoidal coil can be found from the following analysis. In this analysis the Lorentz force on a charge in a current carrying circular wire encompassing the solenoidal structure with the current inducing an axial magnetic field is

$${f}_{r}\,=\,q{v}_{\theta }{B}_{z}$$

where *q* represents the charge, *v*_*θ*_ the angular velocity and *B*_*z*_ the axial magnetic field. For *N* of these charges per unit volume, the number in a volume *dV* is *NdV*. Consequently, the total magnetic force, *dF*, is the sum of the forces on each charge in *dV*. Hence,

$$dF\,=\,NdVq{v}_{\theta }{B}_{z}$$

The term, *Nqv*_*θ*_, is the current density *j*_*θ*_.

Therefore

$$dF\,=\,{j}_{\theta }{B}_{z}dV$$

and so the force per unit volume is *j*_*θ*_*B*_*z*_. For a differential circular wire cross section of thickness *dz* described by 2*πrdr*, the volume is

$$dV\,=\,2\,\pi r\,dr\,dz$$

and so

$$dF\,=\,{j}_{\theta }{B}_{z}\,2\,\pi r\,dr\,dz$$

As a result, for one revolution of the solenoidal circular wire the total force is

21 $$\begin{array}{ccc}F & = & {\int }_{O}^{{\rm{\Delta }}}dz{\int }_{\frac{d}{2}}^{\frac{d}{2}+\lambda }{j}_{\theta }\,{B}_{z}\,2\,\pi r\,dr\\  & = & 2\pi \,{\rm{\Delta }}{\int }_{\frac{d}{2}}^{\frac{d}{2}+\lambda }{j}_{\theta }\,{B}_{z}\,rdr\end{array}$$

Hence, the average force when assuming *e*^−*iωt*^ time dependence is $\hat{F}$ where

22 $$\hat{F}\,=\,\pi {\rm{\Delta }}{\int }_{\frac{d}{2}}^{\frac{d}{2}+\lambda }{B}_{z0}{j}_{\theta 0}\,rdr$$

From (18)

23 $${j}_{\theta 0}\,=\,\,\frac{1}{\lambda }\,\frac{N}{L}\,{I}_{\theta N}$$

By integrating (2), and neglecting the displacement current for $r\, > \,\,\frac{d}{2}$

24 $${B}_{z0}(r)\,=\,{\mu }_{0}{\int }_{\frac{d}{2}}^{\frac{d}{2}+\lambda }{j}_{\theta 0}du\,=\,{\mu }_{0}{j}_{\theta 0}\,\,[\frac{d}{2}\,+\,\lambda \,-\,r]$$

By substituting (23 and 24) into (22) gives

$$\hat{F}\,=\,\frac{{\mu }_{0}\pi {\rm{\Delta }}}{\,{\lambda }^{2}}\,{(\frac{N}{L})}^{2}{|{I}_{\theta N}|}^{2}{\int }_{\frac{d}{2}}^{\frac{d}{2}+\lambda }[\,\frac{d}{2}+\,\lambda -r]rdr$$

$$\hat{F}\,=\,{\mu }_{0}\pi {\rm{\Delta }}{(\frac{N}{L})}^{2}{|{I}_{\theta N}|}^{2}\,\frac{d}{4}\,[1\,+\,\,\frac{2}{3}\,\frac{\lambda }{d}]$$

The average tensile strength on the outer surface of one revolution of the solenoidal coil is

$$\hat{J}\,=\,\frac{\hat{F}}{2\pi {\rm{\Delta }}(\,\frac{d}{2}\,+\,\lambda )}$$

Hence,

25 $$\hat{J}\,=\,\frac{{\mu }_{0}}{\,8{({\rm{\Delta }})}^{2}}{|{I}_{\theta N}|}^{2}\,\frac{d}{(\,\frac{d}{2}+\lambda )}\,[1\,+\,\,\frac{2}{3}\,\frac{\lambda }{d}]$$

## Stacking of Concentric Solenoidal Coils

In this section the approach is to implement stacking of concentric coils to reduce the tensile strength and/or the current flowing in each solenoid while realizing the increased magnetic field result from the sum of the stacking solenoids. For a sequence of concentric solenoids, taken as perfect conductors, the field internal to the innermost solenoidal wall is determined by the boundary condition of the inner surface of that solenoid. This is realized independently of the outer solenoids.

By dividing *λ* in (18) into sections of *λ*/*n* and having each section excited independently by an independent realizable current, then from (18)

$$\begin{array}{c}{I}_{\theta N}\,=\,\frac{L}{N}[{\int }_{\frac{d}{2}}^{\frac{d}{2}+\frac{1}{n}\lambda }{j}_{\theta 0}dr\,+\,{\int }_{\frac{d}{2}+\frac{1}{n}\lambda }^{\frac{d}{2}+\frac{2}{n}\lambda }{j}_{\theta 0}dr\,+\,\ldots \,+\,{\int }_{\frac{d}{2}+\frac{m-1}{n}\lambda }^{\frac{d}{2}+\frac{m}{n}\lambda \,}{j}_{\theta 0}dr\,+\,\ldots \,{\int }_{\frac{d}{2}\,+\frac{n-1}{n}\lambda }^{\frac{d}{2}+\lambda }{j}_{\theta 0}dr]\\ \,\,\,=\,\frac{L}{N}\,{\sum }_{m=1}^{n}{\int }_{\frac{d}{2}+\frac{m-1}{n}\lambda }^{\frac{d}{2}+\frac{m}{n}\lambda \,}{j}_{\theta 0}dr\,=\,{\sum }_{m=1}^{n}{I}_{\theta Nnm}\end{array}$$

where

26 $${I}_{\theta Nnm}\,=\,\frac{L}{N}{\int }_{\frac{d}{2}+\frac{(m-1)}{n}\lambda }^{\frac{d}{2}+\frac{m}{n}\lambda \,}{j}_{\theta 0}\,dr$$

Here, *I*_*θNnm*_ represents the *m*^*th*^ current flowing in the *n*^*th*^ solenoid. Each solenoid has an electrical current independent of the others and must be properly insulated from the rest. The solenoids are in order, concentric to one another with the first solenoid starting at $r\,=\,\,\frac{d}{2}$ and the last one ending $r\,=\,\,\frac{d}{2}\,+\,\lambda$. At this latter distance *B*_*z*_ is assumed to be zero.

## Energy stored internal to a Solenoid

From (4 and 8) and by evaluating *B*_*z*_ at *r* = 0, then

$$\frac{{C}_{1}}{i}\,=\,\frac{{B}_{z0}\,(0)}{\sqrt{{\mu }_{0}\varepsilon }\,}$$

Hence,

$${B}_{z}(r,t)\,=\,{B}_{z0}\,(0)\,{J}_{0}\,(\omega \sqrt{{\mu }_{0}\varepsilon }\,r)\,{e}^{-i\omega t}$$

$${E}_{\theta }(r,t)\,=\,\frac{i\,{B}_{z0}\,(0)}{\sqrt{{\mu }_{0}\varepsilon }\,}\,{J}_{1}\,(\omega \,\sqrt{{\mu }_{0}\varepsilon }\,r)\,{e}^{-i\omega t}$$

The average magnetic energy stored in the solenoid is

27 $$\begin{array}{c}{\hat{ {\mathcal E} }}_{H}\,=\,\frac{1}{4}\,\,{\int }_{V}\,{B}_{z}\,{H}_{z}\,dV\,=\,\frac{\pi \,{B}_{z0}^{2}(0)}{2{\mu }_{0}\,}\,{\int }_{0}^{L}{\int }_{0}^{\frac{d}{2}}r\,{J}_{0}^{2}\,(\omega \sqrt{{\mu }_{0}\varepsilon }\,r)\,dr\,dz\\ \,\,=\,\,\frac{\pi \,{B}_{z0}^{2}\,(0)\,L}{2{\mu }_{0}\,}{\int }_{0}^{d/2}r\,{J}_{0}^{2}\,(\omega \,\sqrt{{\mu }_{0}\varepsilon }r)\,dr\end{array}$$

where *dV* = 2*πrdzdr* and the corresponding electrical energy stored is

28 $${\hat{ {\mathcal E} }}_{E}\,=\,\frac{\pi \,{B}_{z0}^{2}\,(0)\,L}{2{\mu }_{0}\,}{\int }_{0}^{d/2}r\,{J}_{0}^{2}(\omega \,\sqrt{{\mu }_{0}\varepsilon }\,r)\,dr.$$

For $\omega \,\sqrt{{\mu }_{0}\varepsilon }\,\,\frac{d}{2}$ = *δ*_*l*_ from “An Introduction to Linear Analysis” by Kreider, *et al*., p620, 1966^16^ evaluating (27) gives

29 $${\hat{ {\mathcal E} }}_{H}\,=\,{\hat{ {\mathcal E} }}_{E}\,=\,\frac{\pi \,{B}_{z0}^{2}\,(0)\,L}{2{\mu }_{0}\,}(\frac{1}{2}){(\frac{d}{2})}^{2}\,{J}_{0}^{2}(\omega \,\sqrt{{\mu }_{0}\varepsilon }\,\,\frac{d}{2}).$$

## Examples

The two examples to follow are for the cases of the first order field eigenvalue mode, and the fourth order field eigenvalue mode. The potential parameters for assessing the theory presented for large magnetic field solenoidal systems with for example, multi-walled carbon nanotube (MWCNT) – copper composite windings, Subramanian, *et al*.^1^, include the magnetic fields of *B*_*z*0_(0) ranging in multiples of 10 from 2*T* to 2(10)^6^*T*. A point should be made that the world record magnetic field is 100.75*T* as noted earlier^12^. For the presented examples

$$\lambda \,=\,\Delta \,=\,\,50\,\mu m$$

where the MWCNT – copper wire cross section is approximately the size of a corresponding human hair. As already mentioned, Subramanian, *et al*.^1^, have measured for carbon-copper nanotube composite conductors an ampacity of 6(10)^12^*A*/m^2^. Also, they measured a conductivity of *σ* = 4.7(10)^7^ *s*/m and this value will be used in the calculations.

Each magnetic field value considered in the Tables 1 and 2 will give ${j}_{\theta 0},\,{I}_{\theta N},\,\,{V}_{T},\,P,\,\hat{J}\,and\,\frac{{\hat{{\rm E}}}_{H}}{L}.$ Here, from (15, 17, 19, 20, 25 and 29)

$${j}_{\theta 0}\,=\,\,\frac{{B}_{z0}\,(0)\,{J}_{0}\,(\omega \,\sqrt{{\mu }_{0}\varepsilon }\,\,\frac{d}{2})}{{\mu }_{0}\,\lambda }$$

$${I}_{\theta N}\,=\,\,\frac{{\rm{\Delta }}{B}_{z0}(0)\,{J}_{0}\,(\omega \,\sqrt{{\mu }_{0}\varepsilon }\,\frac{d}{2})}{{\mu }_{0}}$$

$${V}_{T}=\,\,\frac{\,{j}_{\theta 0}\pi d}{\sigma }$$

$$P=\,\,\frac{{\rm{\Delta }}\pi d}{\lambda \sigma }{[\frac{{J}_{0}(\omega \sqrt{{\mu }_{0}\varepsilon }\frac{d}{2}){B}_{z0}(0)}{{\mu }_{0}}]}^{2}$$

$$\hat{J}=\,\frac{{\mu }_{0}}{8}{(\frac{{I}_{\theta N}}{{\rm{\Delta }}})}^{2}\frac{d\,}{\frac{d}{2}\,+\,\lambda \,}[1\,+\,\,\frac{2}{3}\,\frac{\lambda }{d}]$$

$$\frac{{\hat{ {\mathcal E} }}_{H}}{L}\,=\,\frac{\pi }{16\,{\mu }_{0}}\,{[d{B}_{z0}(0){J}_{0}(\omega \sqrt{{\mu }_{0}\varepsilon }\frac{d}{2})]}^{2}$$

**Table 1:** *B*_*z*0_(0) − *j*_*θ*0_ − *I*_*θN*_ − *V*_*T*_ (for *d* = 0.1 *mm*, 1 *mm*, and 10 *mm*) − $(P-\hat{J}-\frac{{\hat{ {\mathcal E} }}_{H}}{L})$ (for *d* = 0.1 *mm*, 1 *mm*, and 10 *mm*).

*B*_*z*0_ (0) − *T*	*j*_*θ*0_ − *A*/*m*^2^	*I*_*θN*_ − *A*	*d* − *mm*	*V*_T_ − *V*	*P* − *W*	$\hat{J}$−*GPa*	$\frac{{\hat{{\boldsymbol{ {\mathcal E} }}}}_{{\bf{H}}}}{{\bf{L}}}{\boldsymbol{-}}\frac{{\bf{J}}}{{\bf{L}}}$
2	−13 (10)^9^	32	0.1 1 10	0.0857 0.857 8.57	2.73 27.3 273	0.856 (10)^−4^ 1.24 (10)^−4^ 1.29 (10)^−4^	0.101 (10)^−2^ 0.101 0.101 (10)^2^
20	−13 (10)^10^	320	0.1 1 10	0.857 8.57 85.7	0.273 (10)^3^ 2.73 (10)^3^ 27.3 (10)^3^	0.856 (10)^−2^ 1.24 (10)^−2^ 1.29 (10)^−2^	10.1 (10)^−2^ 10.1 10.1 (10)^2^
200	−13 (10)^11^	3.2 (10)^3^	0.1 1 10	8.57 85.7 857	27.3 (10)^3^ 273 (10)^3^ 2.73 (10)^6^	0.856 1.24 1.29	10.1? 1.01 (10)^3^ 101 (10)^3^
2000	−13 (10)^12^	32 (10)^3^	0.1 1 10	85.7 857 8570	2.73 (10)^6^ 27.3 (10)^6^ 273 (10)^6^	85.6 124 129	1.01 (10)^3^ 101 (10)^3^ 10.1 (10)^6^
20,000	−13 (10)^13^	320 (10)^3^	1	8570	2.73 (10)^6^	12.4 (10)^3^	1.01 (10)^9^
200,000	−13 (10)^14^	3.2 (10)^6^	1	85.7 (10)^3^	273 (10)^6^	1.24 (10)^6^	101 (10)^9^
2,000,000	−13 (10)^15^	32 (10)^6^	1	857 (10)^3^	27.3 (10)^9^	124 (10)^6^	10.1 (10)^12^

**Table 2:** *B*_*z*0_(0) − *j*_*θ*0_ − *I*_*θN*_ − *V*_*T*_ (for *d* = 0.1 *mm*,1  *mm*, and 10 *mm*) −$(P-\hat{J}-\frac{{\hat{ {\mathcal E} }}_{H}}{L})$ (for *d* = 0.1 *mm*, 1 *mm*, and 10 *mm*).

*B*_*z*0_ (0) − *T*	*j*_*θ*0_ − *A*/*m*^2^	*I*θN − *A*	*d* − *mm*	*V*T − *V*	*P* − *W*	$\hat{{\boldsymbol{J}}}$ − *GPa*	$\frac{{\hat{{\boldsymbol{ {\mathcal E} }}}}_{{\boldsymbol{H}}}}{{\boldsymbol{L}}}{\boldsymbol{-}}\frac{{\boldsymbol{J}}}{{\boldsymbol{L}}}$
2	7.05 (10)^9^	17.4	0.1 1 10	0.0471 0.471 4.71	0.812 8.12 81.2	2.53 (10)^−5^ 3.61 (10)^−5^ 3.80 (10)^−5^	0.297 (10)^−3^ 0.0297 2.97
20	7.05 (10)^10^	174	0.1 1 10	0.471 4.71 47.1	81.2 812 8.12 (10)^3^	2.53 (10)^−3^ 3.61 (10)^−3^ 3.80 (10)^−3^	0.0297 2.97 2.97 (10)^2^
200	7.05 (10)^11^	1.74 (10)^3^	0.1 1 10	4.71 47.1 471	8.12 (10)^3^ 81.2 (10)^3^ 812 (10)^3^	0.253 0.361 0.380	2.97 297 29.7 (10)^3^
2000	7.05 (10)^12^	17.4 (10)^3^	0.1 1 10	47.1 471 4710	0.812 (10)^6^ 8.12 (10)^6^ 81.2 (10)^6^	25.3 36.1 38.0	0.297 (10)^3^ 29.7 (10)^3^ 2.97 (10)^6^
20,000	7.05 (10)^13^	174 (10)^3^	1	4710	8.12 (10)^9^	3.8 (10)^3^	2.97 (10)^6^
200,000	7.05 (10)^14^	1.74 (10)^6^	1	47100	81.2 (10)^9^	380 (10)^3^	297 (10)^6^
2,000,000	7.05 (10)^15^	17.4 (10)^6^	1	4710 00	81.2 (10)^12^	38 (10)^6^	29.7 (10)^9^

In viewing some of the parameters an appreciation may be gleaned by noting the availability advertised by the Power Maze Innovation Inc^17^. that offers devices operating at 1–2.5 *GHz* with an output power of 1000 *WCW* with an input/output impedance of 50 Ω.

1. For the first eigenvalue mode

$$\omega \,\sqrt{{\mu }_{0}\varepsilon }\,\frac{d}{2}=3.832$$

or

$$f=\frac{3.832}{\pi \,\sqrt{{\mu }_{0}\varepsilon }\,d}$$

and

$${J}_{0}(3.832)=-\,0.402$$

In this situation

(i) for *ε* = *ε*_*O*_ with *d* = 0.1 *mm* the operating frequency *f* = 3660 *GHz*;
  *d* = 1 *mm*, *f* = 366 *GHz*; and *d* = 10 *mm*,*f* = 36.6 *GHz*.
(ii) for the solenoid filled with titanium material with *ε* = 100*ε*_*O*_ then with *d* = 0.1 *mm*,

*f* = 366 *GHz*; *d* = 1 *mm*, *f* = 36.6 *GHz*; and *d* = 10 *mm*, *f* = 3.66 *GHz*.

When considering prototypes for the first eigenvalue mode, the potential parameters to aid in accessing the theory are presented in Table 1 for large magnetic field solenoidal systems with MWCNT-copper composite windings.

2. For the fourth order eigenvalue mode
  $$\omega \sqrt{{\mu }_{0}\varepsilon }\,\frac{d}{2}=13.3$$
  or
  $$f=\,\frac{13.3}{\pi \sqrt{{\mu }_{0}\varepsilon }\,d}$$
  and
  $${J}_{0}(13.\,3)=0.\,218$$

In this situation

(i) for *ε* = *ε*_*O*_ with *d* = 0.1 *mm*, *f* = 12,700 *GHz*; *d* = 1 *mm*, *f* = 1,270 *GHz*; *d* = 10 *mm*, *f* = 127 *GHz*.
(ii) for *ε* = 100 *ε*_*O*_ with *d* = 0.1 *mm*, *f* = 1,270 *GHz*; *d* = 1 *mm*, *f* = 127 *GHz*; *d* = 10 *mm*, *f* = 12.7 *GHz*.

Table 2 illustrates the potential parameters to assess the theory for large magnetic field prototypes utilizing solenoidal systems with MCWCNT-copper composite windings for the fourth eigenvalue mode.

It should be noted that in the D.C. case *J*_0_(*O*) = 1 that all the quantities in both Tables will be significantly larger than those found in the eigenvalue modes, except for *B*_*z*0_(0) values.

## Conclusions

This theoretical presentation provides parameters to be considered for pursuing multi to mega Tesla magnetic fields using MWCNT-copper composite material. These parameters include the current density, current in one loop, the voltage for one turn of the coil conductor, the power delivered per turn, the tensile strength on the outer surface of each solenoidal coil revolution, solenoid energy stored per unit length. For the first and fourth order eigenvalue modes, when considering prototypes, the Tables present potential parameters for prototypes to assess the theory for large magnetic solenoidal systems with MWCNT-copper composite windings. The solenoidal coils are assumed to be perfect conductors and therefore, the electric field is independent of the magnetic field and approaches zero in the coils. For material such as a fusion target on axis, the electric field is not of consequence because on axis it is zero.

It should be noted from equations (15, 17, 19, 20, 25 and 29) that the trend is for *j*_*θ*0_; *I*_*θN*_; *V*_*T*_; $P;\,\hat{J};\,\frac{{\hat{ {\mathcal E} }}_{H}}{L}\,\,$to decrease for increasing eigenvalues. However, such an increase in eigenvalues, also, gives a significate increase in the operating frequency. For three salient features in studying the factors involved are:

(i) the power provided, *P*, which generates the electrical current limitation in the solenoidal coil and ultimately the magnetic field *B*_*z*0_(0) restriction.
(ii) the tensile strength which presents the mechanical limitation of the solenoid
(iii) the operating frequency which presents the electronic limitation.

The electrical permittivity of carbon appears significantly greater than *ε*_*O*_ and so the solenoidal diameter could be importantly reduced for practical devices. Also, the material inside the solenoid could range above 100 *ε*_*O*_ with say titanium dioxide and so allow the operating frequency to be significantly reduced.

This work indicates through the use of MWCNT-metal composite solenoid windings, very large magnetic fields may be realized and this has potential applications in the miniaturization of magnetic systems.
